# Association between early menopause and adverse cardiovascular events in postmenopausal women: a systematic review and meta-analysis stratified by smoking status and type 2 diabetes mellitus

**DOI:** 10.3389/fpubh.2026.1906440

**Published:** 2026-08-20

**Authors:** Zhongfang Chen, Xinyan Zhang, Yu Chen, Wen Shi, Chaoyue Ma, Xinyue Chen, Shuyu Yao

**Affiliations:** 1 Department of Obstetrics and Gynecology, The First Affiliated Hospital of Zhejiang Chinese Medical University (Zhejiang Provincial Hospital of Chinese Medicine), Hangzhou, Zhejiang, China; 2 The Fifth Clinical Medical College of Zhejiang Chinese Medical University, Hangzhou, Zhejiang, China; 3 The Graduate School, Zhejiang Chinese Medical University, Hangzhou, Zhejiang, China

**Keywords:** cardiovascular events, early menopause, meta-analysis, risk, smoking, T2DM

## Abstract

**Background:**

The impact of early menopause on adverse cardiovascular events may be modified by smoking and type 2 diabetes mellitus (T2DM). This study aims to investigate the effect of early menopause on adverse cardiovascular events in women, with stratification by smoking status and T2DM status.

**Methods:**

Studies were retrieved from four databases (Embase, Web of Science, PubMed, and Cochrane Library) from their inception to January 2026. In this study, early menopause was defined as natural menopause occurring before age 45 years, encompassing both premature menopause (<40 years) and early menopause (40–45 years). This systematic review was registered with PROSPERO (Registration No.: CRD420251238709). Results were presented as hazard ratios (HR) or relative risks (RR) with corresponding 95% confidence intervals (95% CI).

**Results:**

In this study, a total of seven cohort studies involving over 1.77 million postmenopausal women were included. Among non-smoking women, early menopause was associated with a higher risk of heart failure (HF: HR = 1.23, 95% CI = 1.02–1.49, *p* = 0.031, *I*^2^ = 87.9%, *n* = 2) and coronary heart disease (CHD: RR = 1.26, 95% CI = 1.18–1.35, *p* < 0.001, *I*^2^ = 0.0%, *n* = 2). In contrast, among smoking women, we did not find statistically significant evidence of an association between early menopause and either HF (HR = 1.15, 95% CI = 0.88–1.52, *p* = 0.311, *I*^2^ = 73.7%, *n* = 2) or CHD (RR = 1.63, 95% CI = 0.90–2.96, *p* = 0.105, *I*^2^ = 97.2%, *n* = 2). For women without T2DM, early menopause was associated with a higher risk of both HF (HR = 1.19, 95% CI = 1.00–1.43, *p* = 0.050, *I*^2^ = 94.1%, *n* = 2), a borderline finding that should be interpreted with caution, and significantly associated with stroke (HR = 1.11, 95% CI = 1.09–1.13, *p* < 0.001, *I*^2^ = 0.0%, *n* = 2). For women with T2DM, early menopause was associated with a higher risk of HF (HR = 1.29, 95% CI = 1.11–1.50, *p* = 0.001, *I*^2^ = 54.3%, *n* = 2), but not find with stroke risk (HR = 1.11, 95% CI = 0.99–1.25, *p* = 0.062, *I*^2^ = 59.6%, *n* = 2). In both the T2DM and non-T2DM groups, no statistically significant evidence of an association between early menopause and with all-cause mortality was found (T2DM: HR = 1.20, 95% CI = 0.83–1.74, *p* = 0.333, *I*^2^ = 54.3%, *n* = 2; without T2DM: HR = 1.18, 95% CI = 0.94–1.47, *p* = 0.148, *I*^2^ = 53.3%, *n* = 2, respectively).

**Conclusion:**

Early menopause is associated with the risk of adverse cardiovascular events in women, with divergent results by smoking status and T2DM.

**Systematic review registration:**

https://www.crd.york.ac.uk/PROSPERO/view/CRD420251238709; identifier: CRD420251238709.

## Introduction

Cardiovascular disease (CVD) stands as a leading cause of mortality worldwide (1). In 2023, CVD threatened the lives of approximately 19.2 million people worldwide, becoming the leading cause of mortality and disease burden globally (2). Recognized cardiovascular risk factors include obesity, smoking, hypertension, and type 2 diabetes mellitus (T2DM) etc., while estrogen is believed to confer a protective effect on the cardiovascular system, which partly explains the lower incidence of CVD in premenopausal women compared to men of the same age (3, 4). Following menopause (with a decline in estrogen levels), women's cardiovascular risk increases substantially, approximately 2.6 times higher than that observed before menopause (5). Compared with men of the same age, postmenopausal women exhibit a cardiovascular risk that approaches that of men before the age of 60, while after 60, the incidence rate shows a trend of surpassing that of men (6).

Evidence has suggested that early menopause may be associated with adverse cardiovascular events (7). Among women of the same age, those who have undergone menopause exhibit a higher incidence of CVD compared to those who have not. Furthermore, among postmenopausal women, those with early menopause face a greater risk of cardiovascular events than those who experience menopause at a typical age (7). In this study, early menopause was defined as natural menopause occurring before 40 to 45 years of age. However, this inference remains controversial, primarily due to variations in the definition of early menopause across countries and ethnic groups. More importantly, cardiovascular events are influenced by multiple confounding factors, including obesity, smoking, hypertension, and T2DM, which contribute to the ongoing debate regarding the association between early menopause and the risk of CVD (8).

To investigate this issue, the present study aims to collect studies on the correlation between early menopause and cardiovascular events. Obesity, smoking, hypertension and T2DM are established cardiovascular risk factors that may interact with estrogen deficiency, yet no meta-analysis has specifically examined whether they modify this association. Therefore, we conduct stratification by obesity, smoking, hypertension, and T2DM to evaluate whether these risk factors modify the impact of early menopause on female cardiovascular risk.

## Methods

### Search design

This meta-analysis was conducted in accordance with the Preferred Reporting Items for Systematic Reviews of Meta-Analyses (PRISMA) guidelines, with the protocol registered on the PROSPERO platform (Registration No.: CRD420251238709) (9). The protocol prespecified stratified subgroup analyses according to obesity, smoking, hypertension, and T2DM.

### Search strategy

This meta-analysis was conducted by two independent researchers through systematic literature searches, incorporating studies examining the relationship between menopause onset and CVD, adverse cardiovascular factors, and all-cause mortality. A systematic search was conducted in PubMed, Web of Science, Embase, and the Cochrane Library databases for articles published from the inception of each database up to January 2026. Medical subject terms included: (“Menopause,” “Early Menopause,” “Age at Menopause,” “Premature Menopause”) AND (“MACE,” “Cardiovascular Mortality,” “All-cause Mortality,” “Heart Failure,” “Stroke,” “Coronary Disease,” “Myocardial Infarction”). For detailed information, see the Supplementary Table 6. Additionally, formal backward and forward citation searching was performed to minimize the risk of missing relevant literature, including screening reference lists of included studies and tracking their subsequent citations.

### Selection criteria

Studies that fulfilled the following criteria are included: (1) Population: postmenopausal women; (2) Exposure: women with early menopause; (3) Comparison: women with non-early menopause; (4) Outcome: adverse cardiovascular events, including but not limited to heart failure (HF), coronary heart disease (CHD), stroke, and all-cause mortality; (5) Study design: cohort study.

Studies were excluded if any of the following conditions applied: (1) Not published in full; (2) Follow-up duration less than 3 years; (3) Data were not statistically available, i.e., hazard ratios (HR) or relative risks (RR) with 95% confidence intervals (95% CI) were not reported; (4) None of the stratification factors such as obesity, T2DM, hypertension, and smoking were reported. In addition, for multiple publications from the same cohort, we only included the most comprehensive or up-to-date one.

### Data extraction

Data extraction was independently performed by two investigators using a predesigned form. Any discrepancies were resolved through discussion or, if necessary, adjudication by a third investigator. The extracted data included: authors, publication date, study region, cohort name, recruitment time, number of participants, follow-up time, age at enrollment, age at menopause, primary outcome, adjusted factors, effect estimates (HR or RR with corresponding 95% CI) from the maximally adjusted model, and all covariates included in that adjustment model (e.g., age, smoking status, alcohol consumption, menopausal hormone therapy, hypertension, and other relevant covariates).

Given the variation in the definition of “early menopause” across different countries and ethnicities, the menopausal age criterion adopted in this study was based on the reference data from the included literature. According to established literature, premature menopause refers to natural menopause occurring before 40 years of age, primarily caused by premature ovarian failure, whereas early menopause refers to natural menopause occurring between 40 and 45 years of age (10). For the purpose of statistical analysis and in alignment with the most widely recognized standard in academia, both conditions were included in the review and operationally defined as “early menopause” in this study, referring to natural menopause occurring before 45 years of age.

### Study quality assessment

The quality of the included studies was systematically assessed using the Newcastle-Ottawa Scale (NOS). This scale assesses three key dimensions: study selection, cohort comparability, and outcome evaluation, with scores assigned by two independent reviewers on a 9-point scale. The scoring criteria are as follows: studies scoring ≤ 4 are considered low-quality, those scoring 5–6 are moderate-quality, and those scoring ≥7 are high-quality.

### Statistical analysis

All statistical analyses were conducted using STATA 16.0 software. The study assessed the association between age at menopause and cardiovascular events using HR/RR with 95% CI, with stratified analysis according to characteristics of the exposed population such as T2DM, hypertension, and smoking. Given the low incidence of events in the study population, HR and RR were combined under the assumption of rare outcomes. HR/RR > 1 indicated a higher risk of adverse cardiovascular events in the early menopause group, while HR/RR < 1 indicated a lower risk in the early menopause group. Heterogeneity among studies was quantified using the Cochran's Q test and I^2^, with I^2^ used for quantitative analysis: *I*^2^ < 40% was considered no heterogeneity, 40%−60% as mild heterogeneity, and >60% as considerable heterogeneity. Given the inherent heterogeneity across studies, such as variations in demographic characteristics, ethnic diversity, and the definition of early menopause, a random-effects model was therefore employed for data pooling to enhance the robustness of the results. When the number of included studies exceeded 10, sensitivity analyses were conducted by sequentially excluding studies to assess stability of results, with publication bias assessed using Egger's linear regression test. All statistical tests were two-sided, with *p* < 0.05 considered statistically significant.

The reliability of evidence is assessed using the GRADE framework, taking into account factors such as bias risk, inconsistency, indirectness, imprecision, and publication bias.

## Results

### Literature search

**Figure 1:**
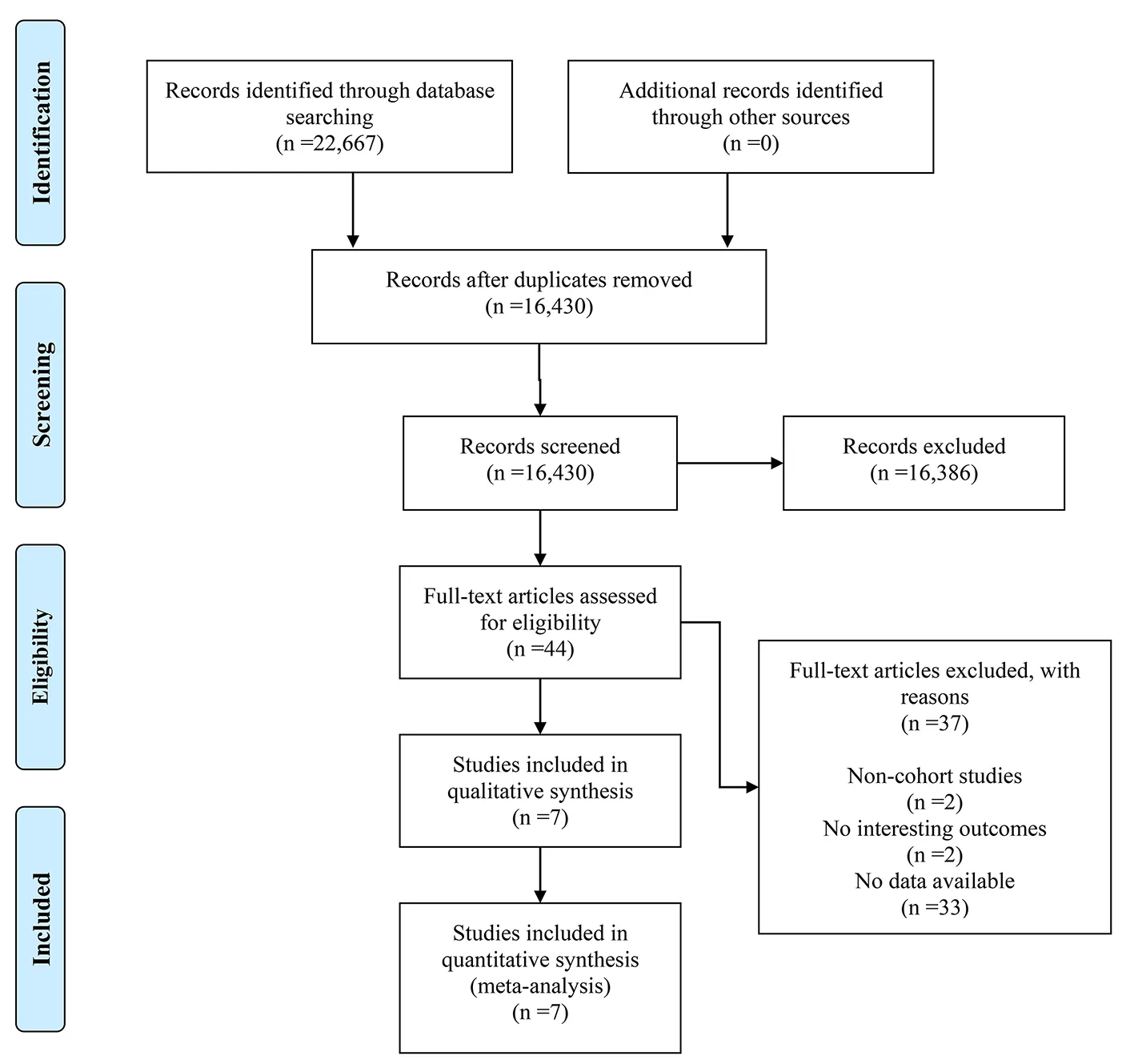
Flow chart representing details of the study selection.

According to the pre-registered search strategy, 22,667 citations were identified, all retrieved from electronic databases; no additional records were uncovered through other sources. After de-duplication using EndNote, 16,430 unique references remained. Title and abstract screening led to the exclusion of 16,386 citations that failed to meet the eligibility criteria. Of the 44 articles advanced to full-text review, 37 were subsequently excluded for the following reasons: (1) non-cohort design (*n* = 2); (2) no outcomes of interest (*n* = 2); (3) no available data (*n* = 33), specifically, these studies investigated the association between early menopause and cardiovascular events but did not present any stratified effect estimates by obesity, smoking, hypertension, or T2DM, and therefore could not contribute to our quantitative synthesis. Consequently, 7 studies were finally included in this systematic review and meta-analysis (11–17). The detailed selection process is presented in Figure 1. Notably, although our pre-registered protocol intended to conduct stratified analyses by obesity and hypertension, no sufficient number of included studies reported available subgroup data for these two variables to perform quantitative synthesis; therefore, only smoking and T2DM were ultimately analyzed as stratification variables.

### Study characteristics

The seven included studies, published between 1999 and 2023, collectively enrolled >1.77 million participants. The primary outcome measures included HF, stroke, CHD, and all-cause mortality, while the exposure factors were T2DM and smoking. Obesity and hypertension were also considered but were not included as exposure factors due to an insufficient number of eligible studies available for analysis. The specific data are detailed in Table 1. Regarding the study regions, they covered Korea, Australia, Sweden, and America. Commonly adjusted factors included age, smoking status, alcohol consumption, body mass index (BMI), hypertension, and menopausal hormone therapy, etc. The detailed information is included in Supplementary Table 1. And effect estimates included HR or RR with corresponding 95% CI, Supplementary Table 2.

**Table 1:** Characteristics of all the studies included in the meta-analysis.

References	Cohort/source (name)	Recruitment time	Number of participants	Follow-up time	Primary outcomes	Exposure factors
Shin et al. (11)	Korean National Health Insurance System	2002–2009	1,401,175	2009–2018	HF	Smoke, obesity, T2DM
Zhu et al. (12)	International Collaboration for a Life Course Approach to Reproductive Health and Chronic Disease Events	1946–2013	301,438	>3 years	stroke, CHD	Smoke
Lee et al. (13)	Korean National Health Insurance System	2009	1,159,405	2009–2019	all-cause mortality, ischemic stroke	Obesity, T2DM
Rahman et al. (14)	Population-based Swedish Mammography Cohort	1997	22,256	1997–2011	HF	Smoke
Yoshida et al. (15)	National Heart, Lung, and Blood Institute Biologic Specimen and Data Repository Information Coordinating Center	1987–2004	9,374	2004–2019	HF, stroke	T2DM
Hu et al. (16)	The Nurses' Health Study cohort	1976	35,616	1976–1994	CHD	Smoke
Asllanaj et al. (17)	Rotterdam Study (RS)	1997–2008	3,650	1999–2008	all-cause mortality	T2DM

HF, heart failure; CHD, coronary heart disease; T2DM, type 2 diabetes mellitus.

For these seven studies, a quality assessment was conducted in this paper, with the evaluation criteria including the selection of the study group, comparability of the cohort, and outcome assessment. The results showed that the scores of all 7 studies were no less than 7 points. The detailed scoring situation is presented in Supplementary Table 3.

### Analysis results

**Figure 2:**
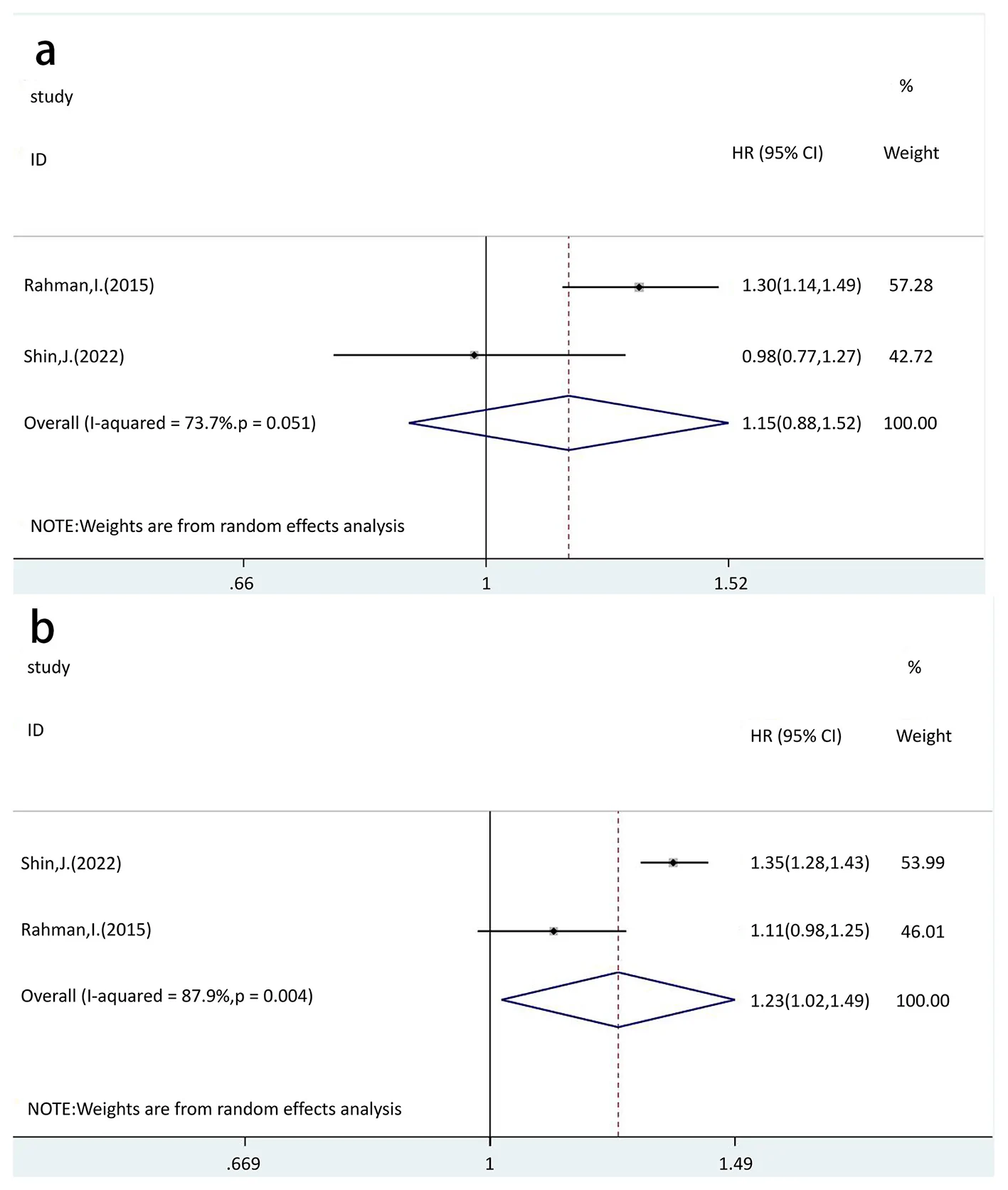
The forest plot demonstrating the impact of early menopause on HF in smoking women [**(a)**, *p* = 0.311] and in non-smoking women [**(b)**, *p* = 0.031]. Weights are from random effects analysis.

**Figure 3:**
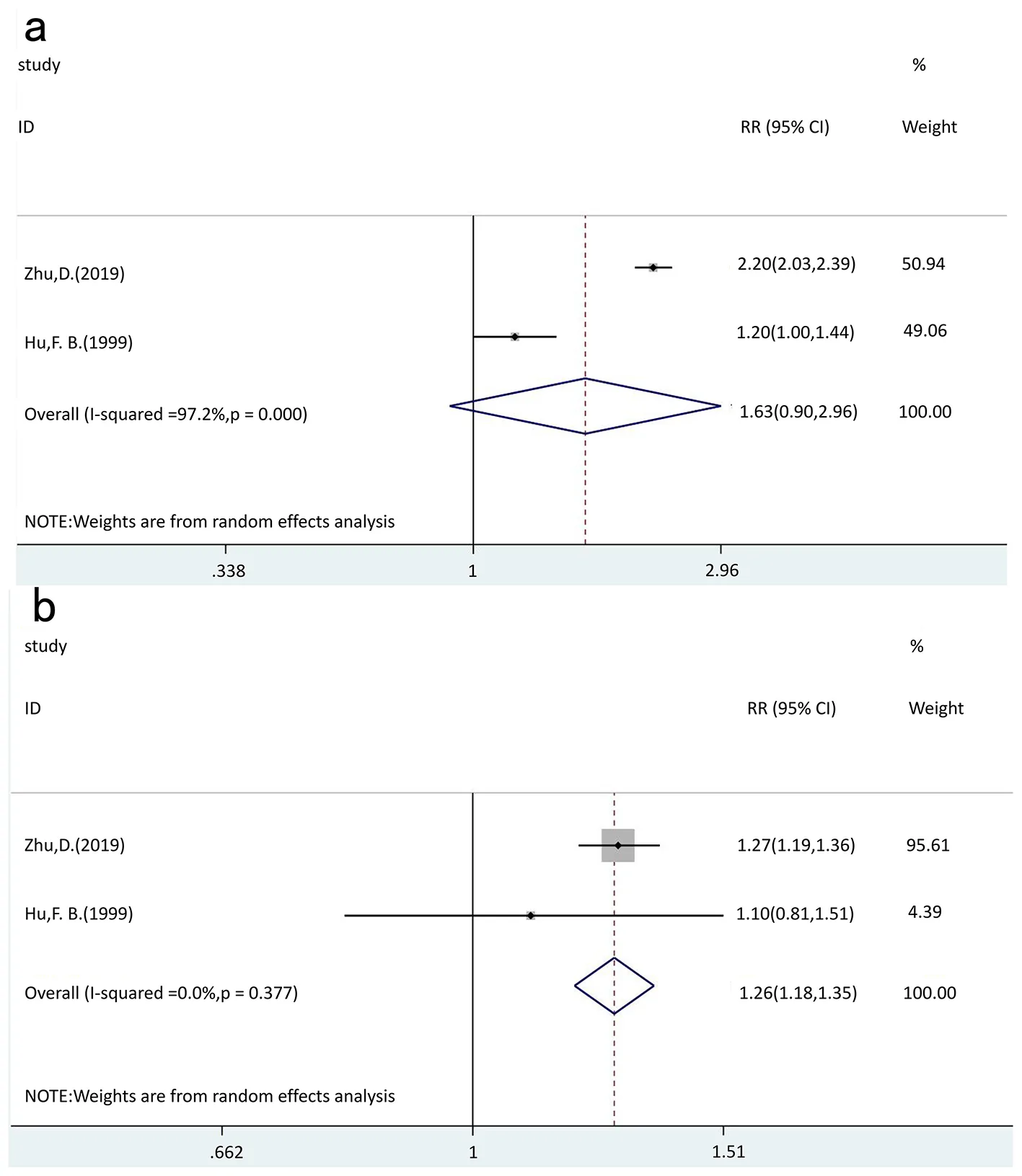
The forest plot demonstrating the impact of early menopause on CHD in smoking women [**(a)**, *p* = 0.105] and in non-smoking women [**(b)**, *p* < 0.001].

For menopausal women who smoke, the incidence of HF did not differ significantly between early menopause and non-early menopause (HR = 1.15, 95% CI = 0.88–1.52, *p* = 0.311, *I*^2^ = 73.7%, *n* = 2); however, among non-smokers, early menopause was associated with a higher risk of HF than in those with non-early menopause (HR = 1.23, 95% CI = 1.02–1.49, *p* = 0.031, *I*^2^ = 87.9%, *n* = 2), as shown in Figure 2. For menopausal women who smoke, the incidence of CHD did not differ significantly between early menopause and non-early menopause (RR = 1.63, 95% CI = 0.90–2.96, *p* = 0.105, *I*^2^ = 97.2%, *n* = 2); however, among non-smokers, early menopause was associated with a higher risk of CHD than in those with non-early menopause (RR = 1.26, 95% CI = 1.18–1.35, *p* < 0.001, *I*^2^ = 0.0%, *n* = 2), as shown in Figure 3. Due to the low incidence of cardiovascular events in the study population, this figure presents a combined estimate of the HR and RR under the rare outcome assumption.

**Figure 4:**
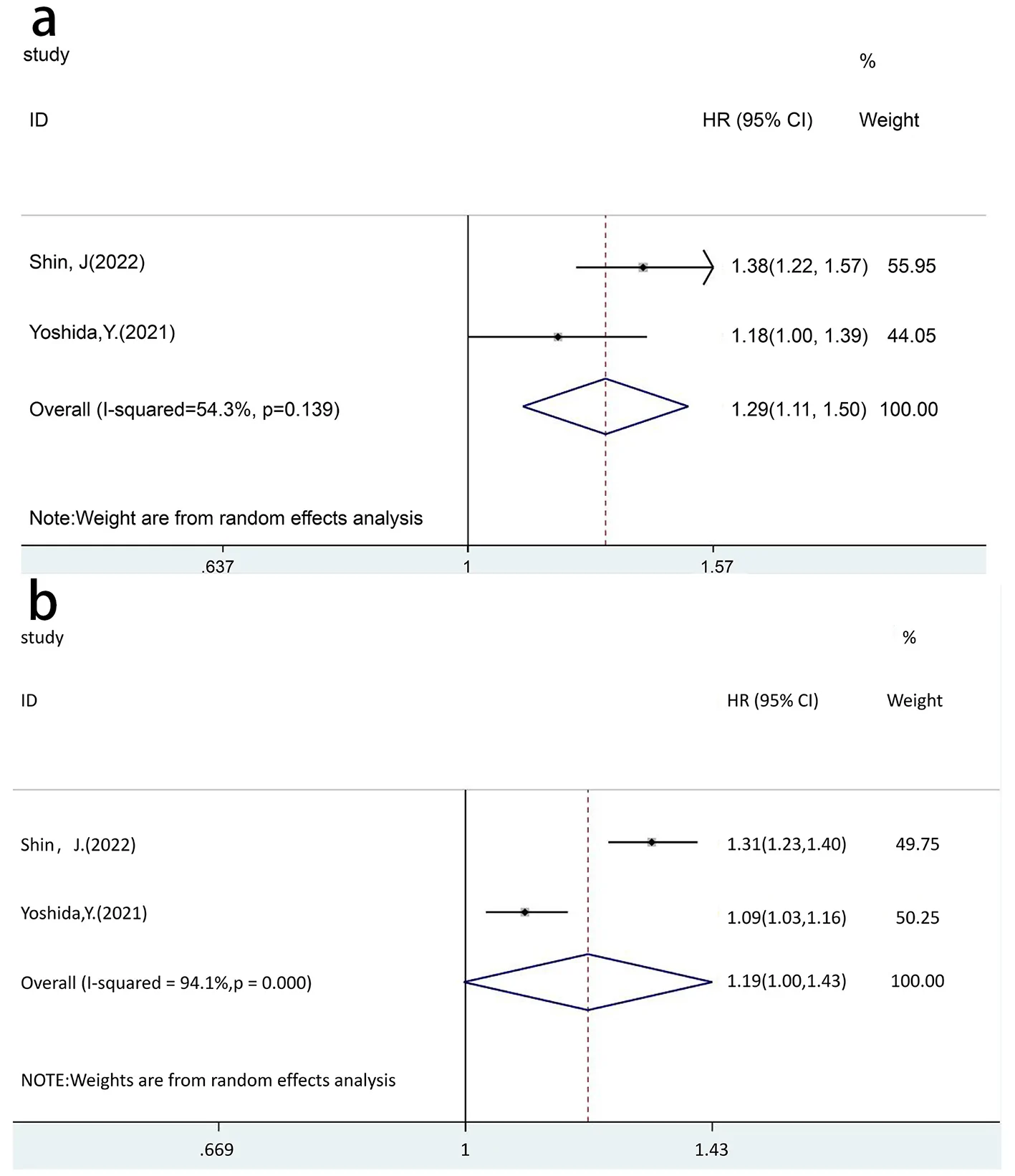
The forest plot demonstrating the impact of early menopause on HF in women with T2DM [**(a)**, *p* = 0.001] and in women without T2DM [**(b)**, *p* = 0.050].

**Figure 5:**
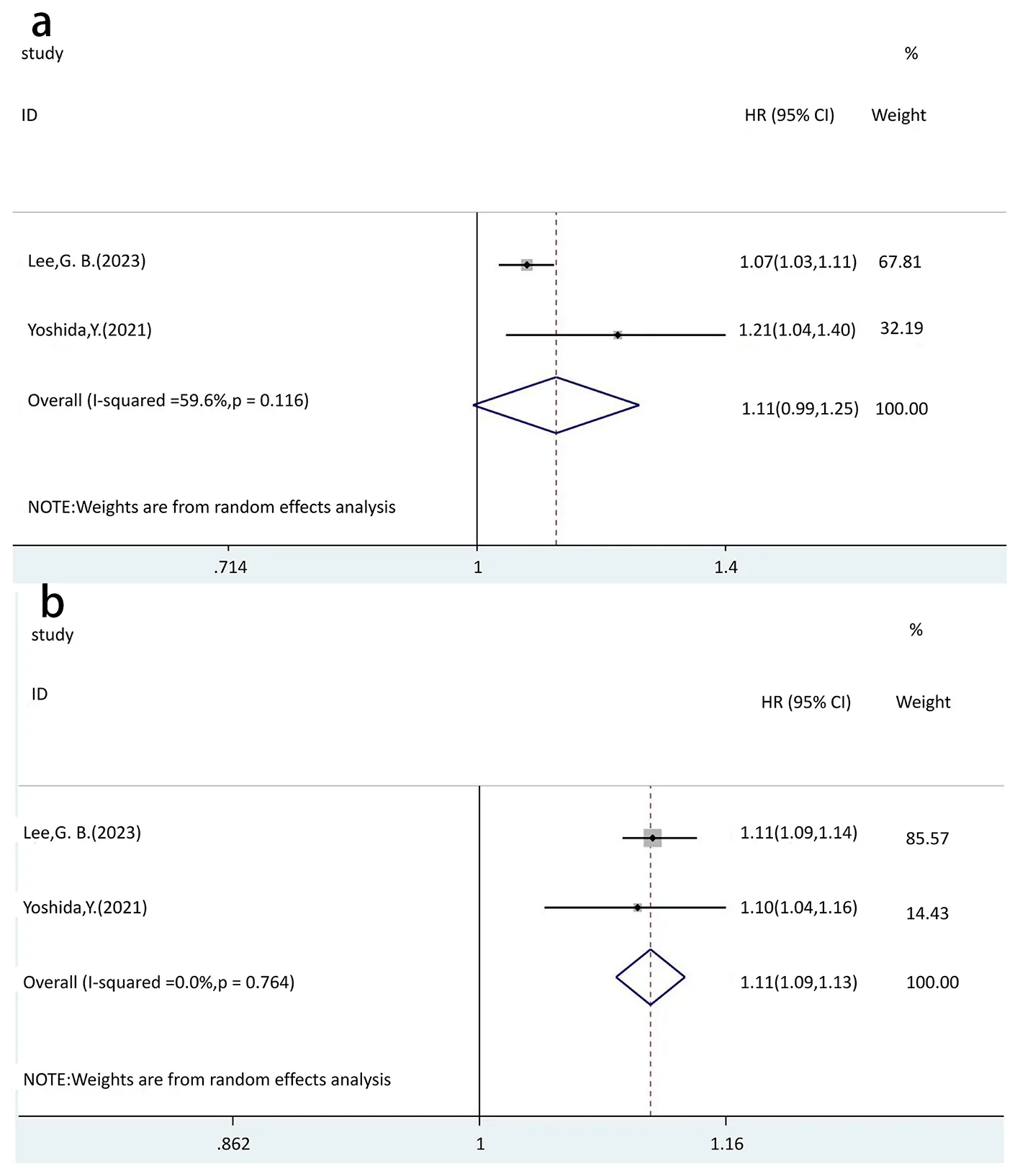
The forest plot demonstrating the impact of early menopause on stroke in women with T2DM [**(a)**, *p* = 0.062] and in women without T2DM [**(b)**, *p* < 0.001].

**Figure 6:**
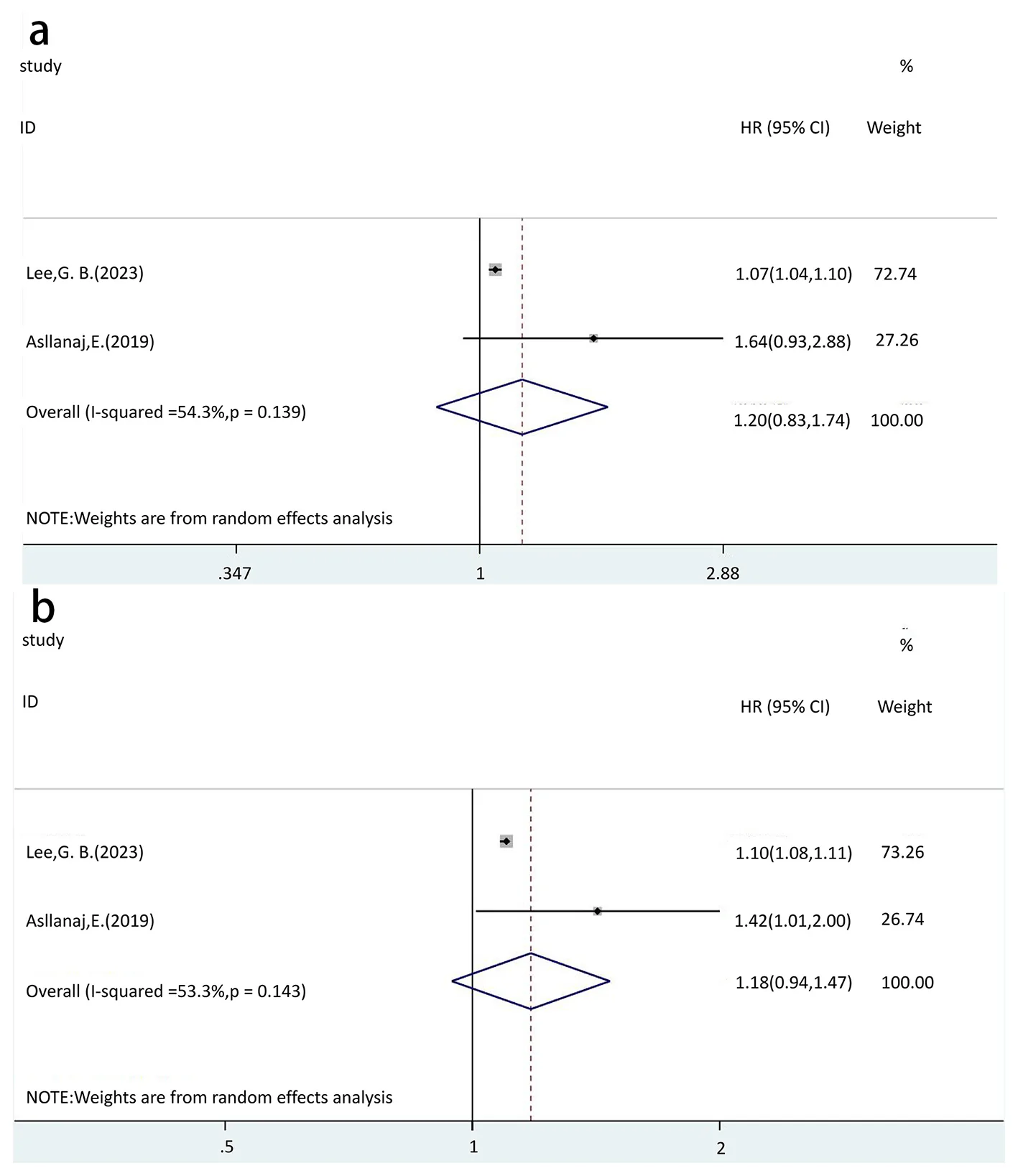
The forest plot demonstrating the impact of early menopause on all-cause mortality in women with T2DM [**(a)**, *p* = 0.333] and in women without T2DM [**(b)**, *p* = 0.148].

Among women with T2DM, early menopause was associated with a higher risk of HF than in those without early menopause (HR = 1.29, 95% CI = 1.11–1.50, *p* = 0.001, *I*^2^ = 54.3%, *n* = 2); similarly, among women without T2DM, early menopause was associated with a higher risk of HF compared to those without early menopause (HR = 1.19, 95% CI = 1.00–1.43, *p* = 0.050, *I*^2^ = 94.1%, *n* = 2), a borderline finding that should be interpreted with caution, as shown in Figure 4. For menopausal women with T2DM, the incidence of stroke did not differ significantly between those with early menopause and those without (HR = 1.11, 95% CI = 0.99–1.25, *p* = 0.062, *I*^2^ = 59.6%, *n* = 2); while for those without T2DM, early menopause was associated with a higher risk of stroke than in those without (HR = 1.11, 95% CI = 1.09–1.13, *p* < 0.001, *I*^2^ = 0.0%, *n* = 2), as shown in Figure 5. For both menopausal women with and without T2DM, all-cause mortality did not differ significantly between those with early menopause and those without (T2DM: HR = 1.20, 95% CI = 0.83–1.74, *p* = 0.333, *I*^2^ = 54.3%, *n* = 2; without T2DM: HR = 1.18, 95% CI = 0.94–1.47, *p* = 0.148, *I*^2^ = 53.3%, *n* = 2, respectively), as shown in Figure 6. Substantial heterogeneity across several stratified analyses may reduce the certainty of our findings.

The results of GRADE showed very low certainty of evidence for the association between early menopause and HF in postmenopausal women who smoke due to serious risk of bias from observational study design and residual confounding, and very low certainty for CHD due to serious risk of bias and serious imprecision, Supplementary Table 4. The results of GRADE also showed low certainty of evidence for the association between early menopause and HF in postmenopausal women with T2DM, and very low certainty for those without T2DM due to serious risk of bias from residual confounding, low certainty for stroke in both groups, and low to very low certainty for all-cause mortality, Supplementary Table 5. Taken together, these findings suggest that smoking status and glycemic status may warrant consideration when evaluating cardiovascular risk in women with early menopause. Given the limited number of studies per stratum, these results should be interpreted as exploratory.

## Discussion

This systematic review and meta-analysis incorporated seven high-quality cohort studies encompassing over 1.77 million participants, examined the associations between early menopause and the risks of HF, stroke, CHD, and all-cause mortality across strata defined by smoking status and T2DM. The principal findings are as follows: among non-smokers or individuals without T2DM, early menopause was associated with a higher risk of HF, CHD, and stroke. Based on this premise, among smokers, no significant association was observed between early menopause and the risks of HF or CHD. In addition, among individuals with T2DM, early menopause was associated with a higher risk of HF, but not find with an increased risk of stroke. In the population with or without T2DM, we did not find statistically significant evidence of an association between early menopause and all-cause mortality.

Early menopause predisposes women to CVD through a multitude of interconnected mechanisms triggered by early estrogen deficiency. Estrogen exerts pleiotropic cardiovascular protective effects, including: preserving endothelial integrity and function by augmenting nitric oxide (NO) bioavailability (18); improving the lipid profile by elevating high-density lipoprotein cholesterol and promoting reverse cholesterol transport, while concurrently reducing low-density lipoprotein oxidation (19); inhibiting vascular smooth muscle cell proliferation and migration (20); attenuating oxidative stress and systemic inflammation (21); and maintaining myocardial energy metabolism and diastolic function (22). The early loss of these protective actions accelerates atherogenesis and heightens susceptibility to cardiovascular events (23).

Specifically concerning HF, estrogen deficiency promotes cardiomyocyte apoptosis, exacerbates myocardial fibrosis, impairs left ventricular diastolic function, and activates the renin-angiotensin-aldosterone system (RAAS) (24–26). These factors collectively increase cardiac workload and contribute to the pathogenesis and progression of HF. Previous meta-analyses have shown that women with premature menopause (< 40 years) have a significantly increased risk of HF by 39% (HR = 1.39), and those with early menopause (< 45 years) have an increased risk of HF by 23% (HR = 1.23) (27). For stroke risk, estrogen deficiency compromises cerebrovascular function by diminishing cerebrovascular reactivity and impairing cerebral autoregulation, while also promoting carotid atherosclerosis through alterations in lipid metabolism (28–30).

Our analysis revealed that early menopause was significantly associated with the risk of HF and CHD, consistent with the mechanisms delineated above. Nevertheless, this association failed to reach statistical significance in the subgroup of women who smoke. This finding suggests that the association between early menopause and cardiovascular risk may differ by smoking status. Several potential explanatory mechanisms warrant consideration.

First, the dominant effect of smoking as a primary risk factor is paramount. Smoking represents a potent independent risk factor for CVD (31), with its atherogenic effects potentially far exceeding those of early menopause. A meta-analysis of prospective studies indicated that the CVD risk for current smokers is approximately twice that of non-smokers (in women: HR = 2.07) (32). More importantly, the detrimental effects of smoking on the cardiovascular system are universal; irrespective of the age at menopause, smoking significantly increases the risk of cardiovascular events (33). Studies have shown that even as few as 1–4 cigarettes per day can elevate the risk of CHD in women by 2- to 3-fold (34). This suggests that the elevated cardiovascular risk associated with smoking may explain why the association between early menopause and cardiovascular events was not observed in this subgroup.

Second, smoking is not only an independent risk factor for cardiovascular adverse events but can also directly induce early menopause through multiple mechanisms. Components of tobacco smoke, such as polycyclic aromatic hydrocarbons (PAHs), exert toxic effects on ovarian function by accelerating follicular depletion (35, 36). Using a rat maternal exposure model, Jurisicova et al. (37) demonstrated that PAHs in cigarette smoke activate the aryl hydrocarbon receptor, inducing the expression of pro-apoptotic proteins Bax and Hrk, which leads to primordial follicle apoptosis and depletion of ovarian reserve, thereby accelerating the onset of menopause. This suggests that early menopause and smoking may not be entirely independent, and that smoking may serve as a risk factor for early menopause. One hypothesis posits that among smokers, women with early menopause may have heavier smoking exposure than those with normal menopausal timing; consequently, the cardiovascular risk factors attributed to early menopause may not be caused by menopause *per se*, but rather by smoking. That is, excessive smoking may both induce early menopause and constitute an independent risk factor for CVD. Therefore, if stratified analysis were conducted according to smoking status, the effect of early menopause might not be observed.

Third, obesity is the most prominent risk factor for HF, particularly among postmenopausal women. Adiposity increases substantially after menopause; a meta-analysis reported an average weight gain of 1.0 kg and an increase in BMI of 1.14 kg/m^2^ compared with premenopausal status (38). This phenomenon is fundamentally attributable to the role of estrogen in maintaining body weight and metabolic homeostasis. For postmenopausal women, early menopause prolongs the duration of estrogen deficiency, which subsequently induces obesity, ultimately predisposing to HF. This null finding may be consistent with the “smoking paradox,” wherein smoking-related factors could obscure the association between early menopause and HF (39–41).

Notably, the analyses examining the interaction between smoking and early menopause in this study exhibited considerable heterogeneity (HF: *I*^2^ = 73.7%; CHD: *I*^2^ = 97.2%), indicating considerable between study variability in smoking definitions, dose assessment methodologies, and population characteristics that may compromise the reliability of effect estimates. Because only two studies were included, this heterogeneity could not be explored through subgroup or meta-regression analyses, and the pooled estimates may not represent a true common effect.

In addition, the number of combined analyses mentioned above is limited, with only two studies meeting the criteria, which introduces the possibility that the observed findings may be due to chance. Therefore, the conclusions of this study should be considered preliminary and cannot be used to infer a definitive causal relationship.

This study observed that T2DM significantly modifies the association between early menopause and cardiovascular events. Specifically, among individuals with T2DM, early menopause was more significantly associated with an elevated risk of HF, but not with an increased risk of stroke. Potential underlying mechanisms include:

T2DM accelerates cardiovascular pathology through vascular endothelial dysfunction and aberrant proliferation of smooth muscle cells, with hyperglycemia-induced inflammatory responses and the formation of advanced glycation end-products further compromising vascular function (42). Concurrently, as diabetic nephropathy progresses to end-stage renal failure, fluid and sodium retention along with the accumulation of uremic toxins precipitate uremic cardiomyopathy, markedly elevating the risk of HF (43). Incidentally, studies have indicated that 40%−50% of patients with HF have concomitant T2DM, and 40% of diabetic patients develop diabetic kidney disease (44). Among individuals with end-stage renal disease, diabetic nephropathy is the predominant etiology, accounting for approximately 44% of new cases in the United States (45). CVD represents the primary cause of death in this population, contributing to over 50% of all fatalities (46), with HF being the most prevalent cardiovascular cause of mortality (47). Additionally, T2DM-related vascular pathology significantly elevates the risk of CHD (48), and myocardial infarction—especially recurrent episodes—secondary to CHD constitutes a major pathogenic mechanism underlying HF (49). Women with early menopause lose the vascular protection conferred by estrogen due to its abrupt decline; when combined with T2DM, this produces a synergistic amplification effect. Estrogen deficiency exacerbates insulin resistance, while T2DM-related oxidative stress further impairs endothelial function—this dual insult significantly accelerates the progression of atherosclerosis and myocardial remodeling. Consequently, among women with T2DM, the impact of early menopause on HF risk is more pronounced, exhibiting an additive effect.

Stroke etiology is predominantly categorized into cardioembolism (25.6%), large-artery atherosclerosis (20.9%), and small-vessel disease (20.5%) (50), with atrial fibrillation (AF) serving as the leading cause of cardioembolic stroke (51). Early menopause has been associated with a higher risk of incident AF in observational studies (27). In recent years, certain glucose-lowering agents, particularly sodium-glucose cotransporter-2 (SGLT2) inhibitors, have demonstrated a protective effect in reducing AF risk among patients with T2DM (52, 53). The present study observed no significant association between early menopause and elevated stroke risk in patients with T2DM. It is possible that concomitant use of glucose-lowering agents that lower AF risk may have influenced this observed null association; however, this hypothesis remains speculative as no data on medication use were collected or analyzed in the present review.

The differential heterogeneity observed in non-T2DM analyses carries important clinical implications, with *I*^2^ = 94.1% for HF analyses and *I*^2^ = 0% for stroke analyses. This discrepancy suggests that the combined effect of non-T2DM and early menopause on stroke risk demonstrates greater consistency across studies, potentially reflecting more clearly defined biological mechanisms.

Concerning all-cause mortality, estrogen deficiency elevates the all-cause mortality rate through the increased risk of the cardiovascular events, as well as potential broad effects on other systems (e.g., immune regulation, bone metabolism, cognitive function) (54). Studies utilized a mouse model of accelerated ovarian failure and found that ovarian hormone deficiency significantly alters peripheral immune cell composition (increased lymphocytes and monocytes), reduces bone density leading to osteoporosis-like changes, and impairs spatial learning and memory function, suggesting that estrogen deficiency increases all-cause mortality risk through multi-system effects (55, 56). A cohort study further confirmed that women experiencing menopause before age 40 have a 25% increased risk of all-cause mortality (HR = 1.25), and those experiencing menopause between ages 40–44 have a 9% increased risk (HR = 1.09) (57). The NHANES I study further confirmed that women with early menopause (< 40 years) have a 53% increased risk of all-cause mortality (HR = 1.53) and a nearly 2-year reduction in lifespan. However, the present study did not observe a statistically significant association between early menopause and all-cause mortality in either the T2DM or non-T2DM population, which may be attributable to insufficient sample size resulting and inadequate follow-up duration resulting in no observed differences (58).

The primary strength of this study lies in the high methodological quality of the included articles, with each achieving a score 7 or higher on the NOS. Furthermore, the analysis is the first systematic effort to concurrently investigate the modifying effects of both smoking and T2DM on the associations between early menopause and multiple cardiovascular events. Additionally, we conducted a comprehensive literature search across four major databases (Embase, Web of Science, PubMed, and Cochrane Library) and registered this review with PROSPERO, ensuring methodological rigor and transparency. This study also has several limitations. First, the relatively small number of studies included, particularly the insufficient sample size for subgroup analyses, suggests that the findings can only serve as a suggestive reference. Moreover, this limited number of studies precluded formal assessment of publication bias, and the potential impact of small-study effects could not be ruled out. Second, significant heterogeneity was observed across studies, primarily attributable to the lack of stratification for key confounders such as variations in menopause definition, smoking status, and glycemic control in patients with T2DM, leading to low credibility of this article and each subgroup should be regarded as exploratory and hypothesis-generating only. Third, early menopause was defined variably across studies. Although premature menopause (natural menopause before 40 years) and early menopause (natural menopause between 40 and 45 years) are conceptually distinct, many included studies used these terms interchangeably or adopted different age cut-offs (< 40 to < 45 years). To standardize the analysis, early menopause was operationally defined as natural menopause occurring before 45 years; however, this underlying variability in source definitions may have introduced residual clinical heterogeneity. Due to the limited number of studies, we were unable to perform sensitivity analyses by specific age cut-offs. Forth, subgroup analyses were conducted within each stratum rather than between strata. Therefore, formal interaction analyses of the effects of smoking or T2DM could not be performed, and no conclusions could be drawn regarding the influence of these factors on the effect estimates. Fifth, the certainty of evidence was rated as low for all outcomes due to serious risk of bias from observational study designs and lack of directly reported stratified event numbers.

## Conclusion

This systematic review and meta-analysis examined the association between early menopause and cardiovascular risk across strata defined by smoking status and T2DM. Among non-smoking and non-T2DM women, early menopause was associated with a higher risk of HF, CHD, and stroke. In the smoking population, we did not find statistically significant evidence of an association between early menopause and either HF or CHD. In women with T2DM, a significant association was observed for HF, but not for stroke. No statistically significant association was found between early menopause and all-cause mortality in either T2DM stratum, although the wide confidence intervals (e.g., HR = 1.20, 95% CI = 0.83–1.74) do not exclude a clinically relevant effect. These findings suggest that the association between early menopause and cardiovascular outcomes may differ according to smoking and T2DM status. However, given the small number of studies contributing to several subgroup analyses and the observed heterogeneity, these results should be considered exploratory.

## Data Availability

The original contributions presented in the study are included in the article/Supplementary material, further inquiries can be directed to the corresponding author.

## Supplementary material

Supplementary Table 1 Detailed characteristics of the studies included in the meta-analysis.

Supplementary Table 2 Characteristics and effect estimates of the studies included in the meta-analysis.

Supplementary Table 3 Quality assessment of the cohort studies included.

Supplementary Table 4 Grade summary of evidence: early menopause and normal menopause for heart failure and coronary heart disease incidence in postmenopausal women (stratified by smoking status).

Supplementary Table 5 Grade summary of evidence: early menopause and normal menopause for heart failure, stroke, and all-cause mortality in postmenopausal women (stratified by type 2 diabetes mellitus status).

Supplementary Table 6 Supplementary search strategy.

Supplementary Table 7 PRISMA 2020 checklist.
