# Supplementary material for: Association between early menopause and adverse cardiovascular events in postmenopausal women: a systematic review and meta-analysis stratified by smoking status and type 2 diabetes mellitus
Source: Front Public Health. 2026 Aug 20;14:1906440. doi: 10.3389/fpubh.2026.1906440 (PMC13538119; doi:10.3389/fpubh.2026.1906440)
Supplement: Supplementary file 1 [file Table_1.docx]

**Supplementary Table 1. Detailed characteristics of the studies included in the meta-analysis.**

| Author | Year | Country/region | Age at enrollment (mean years or range) | Average age at menopause | Menopause category | Adjustment factor |
| --- | --- | --- | --- | --- | --- | --- |
| Shin J | 2022 | Korea | 60 | 50.2 (normal)  36.7 (early) | <40, 40-44, 45-49, ≥50r | age, age at menarche, BMI, smoking status, alcohol consumption, menopausal hormone therapy, T2DM, dyslipidemia, physical activity, income, hypertension, CKD, CHD |
|  |  |  |  |  |  |  |
|  |  |  |  |  |  |  |
|  |  |  |  |  |  |  |
| Zhu D | 2019 | Australia | >32 | 50.2 | <40, 40-44, 45-49, 50-51r, 52-54, ≥55 | age, BMI, menopausal hormone therapy, oral contraception use, educational attainment, hypertension, race |
|  |  |  |  |  |  |  |
| Lee GB | 2023 | Korea | >30 | 50.22 (normal)  36.75 (early) | 30-39, 40-44, 45-49, ≥50r | age, BMI, smoking status, alcohol consumption, menopausal hormone therapy, T2DM, hypertension, physical activity, income, dyslipidemia, glomerular filtration rate |
|  |  |  |  |  |  |  |
|  |  |  |  |  |  |  |
|  |  |  |  |  |  |  |
| Rahman I | 2015 | Sweden | NA | 51 | <45, 46-49, 50-54r, ≥55 | age at menarche, BMI, smoking status, alcohol consumption, oral contraception use, menopausal hormone therapy, parity, educational attainment, T2DM, hypertension, physical activity, family history of MI, history of stroke, history of angina, MI during follow-up, waist circumference |
|  |  |  |  |  |  |  |
| Yoshida Y | 2021 | America | 57.7±8.6 | 45.4 | <45, ≥45r | age, BMI, smoking status, alcohol consumption, ever used hormone therapy, parity, educational attainment, T2DM, mean blood pressure, mean total cholesterol, antihypertensive medication, lipid lowering medication, cohort, race |
|  |  |  |  |  |  |  |
|  |  |  |  |  |  |  |
|  |  |  |  |  |  |  |
| Hu FB | 1999 | America | 30-55 | 50 | <40, 40-44, 45-49, 50-54r, ≥55r | age, smoking status, menopausal hormone therapy, T2DM, systolic blood pressure, AF, cardiovascular disease |
| Asllanaj E | 2019 | America | ≥45 | 40.8 (early)  50.2 (normal)  56.0 (late) | ≤44, 45-50r, ≥55 | age, age at menarche, smoking status, alcohol, hormone therapy, oral contraceptive use, parity, educational attainment, physical activity, cohort |
|  |  |  |  |  |  |  |

NA, not applicable.

r, this refers to the reference value for the control group in each study.

T2DM, type 2 diabetes mellitus; CKD, chronic kidney disease; CHD, coronary heart disease; MI, myocardial infarction; AF, atrial fibrillation; BMI, body mass index.
