# Supplementary material for: Association between early menopause and adverse cardiovascular events in postmenopausal women: a systematic review and meta-analysis stratified by smoking status and type 2 diabetes mellitus
Source: Front Public Health. 2026 Aug 20;14:1906440. doi: 10.3389/fpubh.2026.1906440 (PMC13538119; doi:10.3389/fpubh.2026.1906440)
Supplement: Supplementary file 2 [file Table_2.docx]

**Supplementary Table 2. Characteristics and effect estimates of the studies included in the meta-analysis.**

| Author | Year | Exposure factors | Primary outcomes | study-specific HR/RR | 95% CI |
| --- | --- | --- | --- | --- | --- |
| Shin J | 2022 | smoke | HF | 0.98 | 0.77-1.27 |
|  |  | non-smoke | HF | 1.35 | 1.28-1.43 |
|  |  | T2DM | HF | 1.38 | 1.22-1.57 |
|  |  | non-T2DM | HF | 1.31 | 1.23-1.40 |
| Zhu D | 2019 | smoke | CHD | 2.20 | 2.03-2.39 |
|  |  | non-smoke | CHD | 1.27 | 1.19-1.36 |
| Lee GB | 2023 | T2DM | stroke | 1.07 | 1.03-1.11 |
|  |  | non-T2DM | stroke | 1.11 | 1.09-1.14 |
|  |  | T2DM | all-cause mortality | 1.07 | 1.04-1.10 |
|  |  | non-T2DM | all-cause mortality | 1.10 | 1.08-1.11 |
| Rahman I | 2015 | smoke | HF | 1.30 | 1.14-1.49 |
|  |  | non-smoke | HF | 1.11 | 0.98-1.25 |
| Yoshida Y | 2021 | T2DM | HF | 1.18 | 1.00-1.39 |
|  |  | non-T2DM | HF | 1.09 | 1.03-1.16 |
|  |  | T2DM | stroke | 1.21 | 1.04-1.40 |
|  |  | non-T2DM | stroke | 1.10 | 1.04-1.16 |
| Hu FB | 1999 | smoke | CHD | 1.20 | 1.00-1.44 |
|  |  | non-smoke | CHD | 1.10 | 0.81-1.51 |
| Asllanaj E | 2019 | T2DM | all-cause mortality | 1.64 | 0.93-2.88 |
|  |  | non-T2DM | all-cause mortality | 1.42 | 1.01-2.00 |

HF, heart failure; CHD, coronary heart disease; T2DM, type 2 diabetes mellitus.
