# Supplementary material for: Association between early menopause and adverse cardiovascular events in postmenopausal women: a systematic review and meta-analysis stratified by smoking status and type 2 diabetes mellitus
Source: Front Public Health. 2026 Aug 20;14:1906440. doi: 10.3389/fpubh.2026.1906440 (PMC13538119; doi:10.3389/fpubh.2026.1906440)
Supplement: Supplementary file 3 [file Table_4.docx]

**Supplementary Table 4. Grade summary of evidence: early menopause and normal menopause for heart failure and coronary heart disease incidence in postmenopausal women (stratified by smoking status).**

**Author(s):**

**Question:** Early menopause compared to normal menopause for heart failure and coronary heart disease **Setting:**

**Bibliography:**

| **Certainty assessment** | | | | | | | **№ of patients** | | **Effect** | | **Certainty** | **Importance** |
| --- | --- | --- | --- | --- | --- | --- | --- | --- | --- | --- | --- | --- |
| **№ of studies** | **Study design** | **Risk of bias** | **Inconsistency** | **Indirectness** | **Imprecision** | **Other considerations** | **early menopause** | **normal**  **menopause** | **Relative (95% CI)** | **Absolute (95% CI)** |  |  |

**The incidence of heart failure in postmenopausal women who smoke (assessed with: HR)**

| 2 | non-  randomised  studies | seriousa | not serious | not serious | not serious | none |  |  | **HR 1.15**  (0 .88 to 1 .52) | **1 fewer per**  **1,000**  (from 2 fewer  to 1 fewer) | ⨁◯◯◯  Very lowa | CRITICAL |
| --- | --- | --- | --- | --- | --- | --- | --- | --- | --- | --- | --- | --- |

**The incidence of heart failure in postmenopausal women who non-smoke (assessed with: HR)**

| 2 | non-  randomised  studies | seriousa | not serious | not serious | not serious | none |  |  | **HR 1.23**  (1 .02 to 1 .49) | **1 fewer per**  **1,000**  (from 1 fewer  to 1 fewer) | ⨁◯◯◯  Very lowa | CRITICAL |
| --- | --- | --- | --- | --- | --- | --- | --- | --- | --- | --- | --- | --- |

**The incidence of coronary heart disease in postmenopausal women who smoke (assessed with: RR)**

| 2 | non-  randomised  studies | seriousa | not serious | not serious | not serious | none | 56121/97111  (57 .8%) | 40990/97111  (42 .2%) | **RR 1.63**  (0 .90 to 2 .96) | **266 more per**  **1,000**  (from 42 fewer  to 827 more) | ⨁◯◯◯  Very lowa | CRITICAL |
| --- | --- | --- | --- | --- | --- | --- | --- | --- | --- | --- | --- | --- |

**The incidence of coronary heart disease in postmenopausal women who non-smoke (assessed with: RR)**

| 2 | non-  randomised  studies | not serious | not serious | not serious | not serious | none | 57021/139375  (40 .9%) | 82354/139375  (59 . 1%) | **RR 1.26**  (1 . 18 to 1 .35) | **154 more per**  **1,000**  (from 106  more to 207  more) | ⨁⨁◯◯  Low | CRITICAL |
| --- | --- | --- | --- | --- | --- | --- | --- | --- | --- | --- | --- | --- |

**CI:** confidence interval; **HR:** haz ard ratio; **RR:** risk ratio

**Explanations**

a . When only two studies are inc luded, the I² estimate is highly imprec ise and suffers from extremely low statistical power, making it incapable of acc urately reflecting the true degree of heterogeneity. In this context , the purpose of generating a forest plot is not to force a pooled analysis to derive a definitive overall effect siz e , but rather to visually highlight the disc repanc ies or differences between the two studies . This provides visual evidence to support downgrading for 'inconsistency' in the GRADE assessment , while ensuring the presentation of evidence remains transparent and complete .
