# Supplementary material for: Association between early menopause and adverse cardiovascular events in postmenopausal women: a systematic review and meta-analysis stratified by smoking status and type 2 diabetes mellitus
Source: Front Public Health. 2026 Aug 20;14:1906440. doi: 10.3389/fpubh.2026.1906440 (PMC13538119; doi:10.3389/fpubh.2026.1906440)
Supplement: Supplementary file 4 [file Table_5.docx]

**Supplementary Table 5. Grade summary of evidence: early menopause and normal menopause for heart failure, stroke, and all-cause mortality in postmenopausal women (stratified by type 2 diabetes mellitus status).**

**Author(s):**

**Question:** Early menopause compared to normal menopause for heart failure and stroke and all-cause mortality **Setting:**

**Bibliography:**

**Certainty assessment	№ of patients	Effect**

**Certainty	Importance**

**№ of**

**normal	Relative**

**menopause	(95% CI)**

**Absolute**

**(95% CI)**

**studies	Study design	Risk of bias	Inconsistency	Indirectness	Imprecision	Other considerations	early menopause**

**The incidence of heart failure in postmenopausal women who with type 2 diabetes mellitus (assessed with: HR)**

| 2 | non-  randomised  studies | not serious | not serious | not serious | not serious | none |  |  | **HR 1.29**  (1 . 11 to 1 .50) | **1 fewer per**  **1,000**  (from 2 fewer  to 1 fewer) | ⨁⨁◯◯  Low | CRITICAL |
| --- | --- | --- | --- | --- | --- | --- | --- | --- | --- | --- | --- | --- |

**The incidence of heart failure in postmenopausal women who without type 2 diabetes mellitus (assessed with: HR)**

| 2 | non-  randomised  studies | seriousa | not serious | not serious | not serious | none |  |  | **HR 1.19**  (1 .00 to 1 .43) | **1 fewer per**  **1,000**  (from 1 fewer  to 1 fewer) | ⨁◯◯◯  Very lowa | CRITICAL |
| --- | --- | --- | --- | --- | --- | --- | --- | --- | --- | --- | --- | --- |

**The incidence of stroke in postmenopausal women who with type 2 diabetes mellitus (assessed with: HR)**

| 2 | non-  randomised  studies | not serious | not serious | not serious | not serious | none |  |  | **HR 1.11**  (0 .99 to 1 .25) | **1 fewer per**  **1,000**  (from 1 fewer  to 1 fewer) | ⨁⨁◯◯  Low | CRITICAL |
| --- | --- | --- | --- | --- | --- | --- | --- | --- | --- | --- | --- | --- |

**The incidence of stroke in postmenopausal women who without type 2 diabetes mellitus (assessed with: HR)**

| 2 | non-  randomised  studies | not serious | not serious | not serious | not serious | none |  |  | **HR 1.11**  (1 .09 to 1 . 13) | **1 fewer per**  **1,000**  (from 1 fewer  to 1 fewer) | ⨁⨁◯◯  Low | CRITICAL |
| --- | --- | --- | --- | --- | --- | --- | --- | --- | --- | --- | --- | --- |

**The incidence of all-cause mortality in postmenopausal women who with type 2 diabetes mellitus (assessed with: HR)**

| 2 | non-  randomised  studies | not serious | not serious | not serious | not serious | none |  |  | **HR 1.20**  (0 .83 to 1 .74) | **1 fewer per**  **1,000**  (from 2 fewer  to 1 fewer) | ⨁⨁◯◯  Low | IMPORTANT |
| --- | --- | --- | --- | --- | --- | --- | --- | --- | --- | --- | --- | --- |

**The incidence of all-cause mortality in postmenopausal women who without type 2 diabetes mellitus (assessed with: HR)**

| 2 | non-  randomised  studies | not serious | not serious | not serious | not serious | none |  |  | **HR 1.18**  (0 .94 to 1 .47) | **1 fewer per**  **1,000**  (from 1 fewer  to 1 fewer) | ⨁⨁◯◯  Low | IMPORTANT |
| --- | --- | --- | --- | --- | --- | --- | --- | --- | --- | --- | --- | --- |

**CI:** confidence interval; **HR:** haz ard ratio

**Explanations**

a .
