# Supplementary material for: Association between early menopause and adverse cardiovascular events in postmenopausal women: a systematic review and meta-analysis stratified by smoking status and type 2 diabetes mellitus
Source: Front Public Health. 2026 Aug 20;14:1906440. doi: 10.3389/fpubh.2026.1906440 (PMC13538119; doi:10.3389/fpubh.2026.1906440)
Supplement: Supplementary file 5 [file Table_6.docx]

**Supplementary** **Search Strategy.**

**Pubmed**

| Search number | Query | Search Details | Results |
| --- | --- | --- | --- |
| 1 | (MACE OR cardiovascular disease OR CVD OR cardiovascular disease OR cardiovascular events OR adverse cardiovascular OR cardiovascular system OR cardiac events OR all-cause death OR cardiovascular death OR all-cause mortality OR cardiovascular mortality OR stroke OR myocardial infarction OR heart failure OR coronary disease OR myocardial infarction) | "myristica"[MeSH Terms] OR "myristica"[All Fields] OR "mace"[All Fields] OR ("cardiovascular diseases"[MeSH Terms] OR ("cardiovascular"[All Fields] AND "diseases"[All Fields]) OR "cardiovascular diseases"[All Fields] OR ("cardiovascular"[All Fields] AND "disease"[All Fields]) OR "cardiovascular disease"[All Fields]) OR "CVD"[All Fields] OR ("cardiovascular diseases"[MeSH Terms] OR ("cardiovascular"[All Fields] AND "diseases"[All Fields]) OR "cardiovascular diseases"[All Fields] OR ("cardiovascular"[All Fields] AND "disease"[All Fields]) OR "cardiovascular disease"[All Fields]) OR (("cardiovascular system"[MeSH Terms] OR ("cardiovascular"[All Fields] AND "system"[All Fields]) OR "cardiovascular system"[All Fields] OR "cardiovascular"[All Fields] OR "cardiovasculars"[All Fields]) AND ("event"[All Fields] OR "event s"[All Fields] OR "events"[All Fields])) OR (("adverse"[All Fields] OR "adversely"[All Fields] OR "adverses"[All Fields]) AND ("cardiovascular system"[MeSH Terms] OR ("cardiovascular"[All Fields] AND "system"[All Fields]) OR "cardiovascular system"[All Fields] OR "cardiovascular"[All Fields] OR "cardiovasculars"[All Fields])) OR ("cardiovascular system"[MeSH Terms] OR ("cardiovascular"[All Fields] AND "system"[All Fields]) OR "cardiovascular system"[All Fields]) OR ("cardiovascular diseases"[MeSH Terms] OR ("cardiovascular"[All Fields] AND "diseases"[All Fields]) OR "cardiovascular diseases"[All Fields] OR ("cardiac"[All Fields] AND "events"[All Fields]) OR "cardiac events"[All Fields]) OR ("all-cause"[All Fields] AND ("death"[MeSH Terms] OR "death"[All Fields] OR "deaths"[All Fields])) OR (("cardiovascular system"[MeSH Terms] OR ("cardiovascular"[All Fields] AND "system"[All Fields]) OR "cardiovascular system"[All Fields] OR "cardiovascular"[All Fields] OR "cardiovasculars"[All Fields]) AND ("death"[MeSH Terms] OR "death"[All Fields] OR "deaths"[All Fields])) OR ("all-cause"[All Fields] AND ("mortality"[MeSH Terms] OR "mortality"[All Fields] OR "mortalities"[All Fields] OR "mortality"[MeSH Subheading])) OR (("cardiovascular system"[MeSH Terms] OR ("cardiovascular"[All Fields] AND "system"[All Fields]) OR "cardiovascular system"[All Fields] OR "cardiovascular"[All Fields] OR "cardiovasculars"[All Fields]) AND ("mortality"[MeSH Terms] OR "mortality"[All Fields] OR "mortalities"[All Fields] OR "mortality"[MeSH Subheading])) OR ("stroke"[MeSH Terms] OR "stroke"[All Fields] OR "strokes"[All Fields] OR "stroke s"[All Fields]) OR ("myocardial infarction"[MeSH Terms] OR ("myocardial"[All Fields] AND "infarction"[All Fields]) OR "myocardial infarction"[All Fields]) OR ("heart failure"[MeSH Terms] OR ("heart"[All Fields] AND "failure"[All Fields]) OR "heart failure"[All Fields]) OR ("coronary disease"[MeSH Terms] OR ("coronary"[All Fields] AND "disease"[All Fields]) OR "coronary disease"[All Fields]) OR ("myocardial infarction"[MeSH Terms] OR ("myocardial"[All Fields] AND "infarction"[All Fields]) OR "myocardial infarction"[All Fields]) | 4,288,089 |
| 2 | (premenopausal OR early menopause OR perimenopausal OR premature menopause OR menopause OR menopausal) | "premenopausal"[All Fields] OR "premenopausally"[All Fields] OR "premenopausals"[All Fields] OR "premenopause"[MeSH Terms] OR "premenopause"[All Fields] OR "premenopausic"[All Fields] OR ("menopause, premature"[MeSH Terms] OR ("menopause"[All Fields] AND "premature"[All Fields]) OR "premature menopause"[All Fields] OR ("early"[All Fields] AND "menopause"[All Fields]) OR "early menopause"[All Fields]) OR ("perimenopausal"[All Fields] OR "perimenopausally"[All Fields] OR "perimenopause"[MeSH Terms] OR "perimenopause"[All Fields]) OR ("menopause, premature"[MeSH Terms] OR ("menopause"[All Fields] AND "premature"[All Fields]) OR "premature menopause"[All Fields] OR ("premature"[All Fields] AND "menopause"[All Fields])) OR ("menopause"[MeSH Terms] OR "menopause"[All Fields] OR "menopausal"[All Fields] OR "menopaused"[All Fields] OR "menopauses"[All Fields]) OR ("menopause"[MeSH Terms] OR "menopause"[All Fields] OR "menopausal"[All Fields] OR "menopaused"[All Fields] OR "menopauses"[All Fields]) | 117,712 |
| 3 | #1 AND #2 | #1 AND #2 | 14,229 |

**Web of Science**

| Search number | Query | Search Details | Results |
| --- | --- | --- | --- |
| 1 | TS=((cardiovascular events) OR (MACE) OR (CVD) OR (cardiovascular disease) OR (cardiovascular events) OR (adverse cardiovascular) OR (cardiovascular system) OR (cardiac events) OR (all-cause death) OR (cardiovascular death) OR (stroke) OR (myocardial infarction) OR (heart failure) OR (coronary disease) OR (myocardial infarction)) | TS=((cardiovascular events) OR (MACE) OR (CVD) OR (cardiovascular disease) OR (cardiovascular events) OR (adverse cardiovascular) OR (cardiovascular system) OR (cardiac events) OR (all-cause death) OR (cardiovascular death) OR (stroke) OR (myocardial infarction) OR (heart failure) OR (coronary disease) OR (myocardial infarction)) | 13,079 |
| 2 | TS=((premenopausal) OR (early menopause） OR (perimenopausal) OR (premature menopause) OR (menopause) OR (menopausal)) | TS=((premenopausal) OR (early menopause） OR (perimenopausal) OR (premature menopause) OR (menopause) OR (menopausal)) | 56,881 |
| 3 | #1 AND #2 | #1 AND #2 | 5,157 |

**EMBASE**

| Search number | Query | Search Details | Results |
| --- | --- | --- | --- |
| 1 | (((((((((((((('mace'/exp OR mace OR 'cardiovascular'/exp OR cardiovascular) AND ('disease'/exp OR disease) OR cvd OR 'cardiovascular'/exp OR cardiovascular) AND ('disease'/exp OR disease) OR 'cardiovascular'/exp OR cardiovascular) AND events OR adverse) AND ('cardiovascular'/exp OR cardiovascular) OR 'cardiovascular'/exp OR cardiovascular) AND system OR 'cardiac'/exp OR cardiac) AND events OR 'all cause') AND ('death'/exp OR death) OR 'cardiovascular'/exp OR cardiovascular) AND ('death'/exp OR death) OR 'all cause') AND ('mortality'/exp OR mortality) OR 'cardiovascular'/exp OR cardiovascular) AND ('mortality'/exp OR mortality) OR 'stroke'/exp OR stroke OR myocardial) AND ('infarction'/exp OR infarction) OR 'heart'/exp OR heart) AND ('failure'/exp OR failure) OR coronary) AND ('disease'/exp OR disease) OR myocardial) AND ('infarction'/exp OR infarction) | (((((((((((((('mace'/exp OR mace OR 'cardiovascular'/exp OR cardiovascular) AND ('disease'/exp OR disease) OR cvd OR 'cardiovascular'/exp OR cardiovascular) AND ('disease'/exp OR disease) OR 'cardiovascular'/exp OR cardiovascular) AND events OR adverse) AND ('cardiovascular'/exp OR cardiovascular) OR 'cardiovascular'/exp OR cardiovascular) AND system OR 'cardiac'/exp OR cardiac) AND events OR 'all cause') AND ('death'/exp OR death) OR 'cardiovascular'/exp OR cardiovascular) AND ('death'/exp OR death) OR 'all cause') AND ('mortality'/exp OR mortality) OR 'cardiovascular'/exp OR cardiovascular) AND ('mortality'/exp OR mortality) OR 'stroke'/exp OR stroke OR myocardial) AND ('infarction'/exp OR infarction) OR 'heart'/exp OR heart) AND ('failure'/exp OR failure) OR coronary) AND ('disease'/exp OR disease) OR myocardial) AND ('infarction'/exp OR infarction) | 554,254 |
| 2 | ((premenopausal OR early) AND menopause OR perimenopausal OR premature) AND menopause OR menopause OR 'menopause' | ((premenopausal OR early) AND menopause OR perimenopausal OR premature) AND menopause OR menopause OR 'menopause' | 102,186 |
| 3 | #1 AND #2 | #1 AND #2 | 1,188 |

**Cochrane**

| Search number | Query | Search Details | Results |
| --- | --- | --- | --- |
| 1 | MACE OR cardiovascular disease OR CVD OR cardiovascular disease OR cardiovascular events OR adverse cardiovascular OR cardiovascular system OR cardiac events OR all-cause death OR cardiovascular death OR all-cause mortality OR cardiovascular mortality OR stroke OR myocardial infarction OR heart failure OR coronary disease OR myocardial infarction | (MACE OR cardiovascular disease OR CVD OR cardiovascular disease OR cardiovascular events OR adverse cardiovascular OR cardiovascular system OR cardiac events OR all-cause death OR cardiovascular death OR all-cause mortality OR cardiovascular mortality OR stroke OR myocardial infarction OR heart failure OR coronary disease OR myocardial infarction):ti,ab,kw | 244,354 |
| 2 | premenopausal OR early menopause OR perimenopausal OR premature menopause OR menopause OR menopausal | (premenopausal OR early menopause OR perimenopausal OR premature menopause OR menopause OR menopausal):ti,ab,kw | 22,878 |
| 3 | #1 AND #2 | #1 AND #2 | 2,093 |
