# Supplementary material for: Association between early menopause and adverse cardiovascular events in postmenopausal women: a systematic review and meta-analysis stratified by smoking status and type 2 diabetes mellitus
Source: Front Public Health. 2026 Aug 20;14:1906440. doi: 10.3389/fpubh.2026.1906440 (PMC13538119; doi:10.3389/fpubh.2026.1906440)
Supplement: Supplementary file 7 [file Table_3.doc]

**Supplementary Table 3. Quality assessment of the cohort studies included.**

| Author, year | Selection (Out of 4) | | | | Comparability  (Out of 2) | Outcomes (Out of 3) | | | Total  (Out of 9) |
| --- | --- | --- | --- | --- | --- | --- | --- | --- | --- |
|  | Representativeness of exposed cohort | Selection of non exposed cohort | Ascertainment  of exposure | Outcome not present at the start of the study |  | Assessment of outcomes | Length of follow-up | Adequacy of follow up of cohorts |  |
| Shin J, 2022 | 1 | 1 | 1 | 1 | 2 | 1 | 1 | 1 | 9 |
| Zhu D, 2019 | 1 | 1 | 1 | 1 | 1 | 1 | 1 | 1 | 8 |
| Lee GB, 2023 | 0 | 1 | 1 | 1 | 1 | 1 | 1 | 1 | 7 |
| Rahman I, 2015 | 1 | 1 | 1 | 1 | 1 | 1 | 1 | 1 | 8 |
| Yoshida Y, 2021 | 1 | 1 | 1 | 1 | 1 | 1 | 0 | 1 | 8 |
| Hu FB, 1999 | 1 | 1 | 1 | 1 | 0 | 1 | 1 | 1 | 7 |
| Asllanaj E, 2019 | 1 | 1 | 1 | 1 | 1 | 1 | 1 | 1 | 8 |

The cohort studies were assessed by the Newcastle-Ottawa Quality Assessment Scale (NOS) checklist.
